# Pyoderma gangrenosum, acne, and suppurative hidradenitis (PASH) syndrome: a single-institution case series with a focus on management

**DOI:** 10.1007/s00403-024-03125-7

**Published:** 2024-06-15

**Authors:** Allison Yan, Matthew Gallardo, Andrei Savu, Benjamin Kaffenberger

**Affiliations:** 1 The Ohio State University College of Medicine, Columbus, OH USA; 2 Department of Dermatology, The Ohio State University Wexner Medical Center, 1328 Dublin Rd. Suite 100, Columbus, OH 43212 USA

**Keywords:** PASH syndrome, Pyoderma gangrenosum, Hidradenitis suppurativa, Acne

## Abstract

**Background:**

Pyoderma gangrenosum, acne, and suppurative hidradenitis (PASH) syndrome is a rare condition characterized by clinical features of all three dermatologic conditions. The management of PASH syndrome is difficult, with no consensus on treatment guidelines. Since PASH syndrome can increase morbidity and adversely impact quality of life, better characterization of effective therapies is needed.

**Methods:**

A retrospective cohort study was conducted to identify all patients with pyoderma gangrenosum (PG) treated at The Ohio State University Wexner Medical Center between 2015 and 2021. PG diagnosis was confirmed via PARACELSUS score. Subsequent chart review identified eight patients with concomitant hidradenitis suppurativa (HS) and acne who were clinically diagnosed with PASH syndrome.

**Results:**

Eight patients were clinically diagnosed with PASH syndrome based on their clinical presentation at our institution. Seven patients had failed some type of medical therapy prior to presentation, including topical corticosteroids, oral corticosteroids, oral antibiotics, and biologics. One patient had also tried surgical drainage at an outside institution. Six patients were effectively treated with biologics, usually in combination with other therapies. One patient experienced improvement of her skin lesions after diagnosis and treatment of her underlying hematologic malignancy.

**Conclusions:**

Medical management with biologics in combination with corticosteroids and/or antibiotics was effective in the management of most patients. Diagnosis and treatment of an underlying condition should be prioritized in refractory cases. If workup is negative, surgical management may be considered. Further investigation with a greater number of patients is required to develop management guidelines for PASH syndrome.

## Introduction

Pyoderma gangrenosum, acne, and suppurative hidradenitis (PASH) syndrome is a rare autoinflammatory syndrome characterized by the clinical symptoms of all three dermatologic conditions [1]. While the pathogenesis of PASH syndrome has not been clearly elucidated, it is thought to be due to dysregulation of the innate immune response resulting in neutrophil-mediated inflammation [2]. It disproportionately affects younger males, with a nearly 2:1 male-to-female ratio and a median age of 34 years at diagnosis [3]. Acne most commonly presents first, followed by HS and then PG; patients with PASH syndrome often have more severe lesions for all three of these diseases [2, 4]. As PASH syndrome can adversely impact the physical and emotional well-being of affected patients, effective management is critical to reduce morbidity and improve quality of life.

The therapeutic management of PASH syndrome is challenging, with no consensus on best management principles. There have been reported successful therapeutic options, with corticosteroids, biologic medications, systemic antibiotics, and other immune modifying or suppressing drugs most impactful along with wound management and healthy lifestyle changes [2]. Anti-TNF agents such as infliximab and adalimumab are the most prescribed medications to treat PASH syndrome, usually with varying success [2, 5, 6]. Efficacy of anti-TNF agents in PASH resolution may be related to the underlying connection between pyoderma gangrenosum (PG), hidradenitis suppurativa (HS), and inflammatory bowel disease (IBD), and can optimally be used to manage both conditions with only one medication [7, 8].

This single-institution case series describes the initial presentation of 8 patients with PASH syndrome, laboratory findings, treatment efficacy, and comorbidities laboratory studies.

## Methods

A retrospective cohort study was conducted to identify all patients diagnosed with pyoderma gangrenosum at the Ohio State University Wexner Medical Center and seen by the principal investigator between 2015 and 2021. PG diagnoses were confirmed via PARACELSUS scores. Subsequently, patients who had concomitant diagnoses of acne and HS were identified. An official diagnosis of PASH syndrome was documented in 8 patients. Failed treatments were defined as treatments that did not result in wound closure after at least six months, or treatments that the patient stopped for any reason at any time and did not result in wound closure. Effective treatments were defined as treatments that resulted in partial or full closure of skin lesions on initial presentation to our institution after any time period.

### Results/Cases

Our patients were primarily white (75%) females (62.5%) with a mean age of 37 (range 18–59) at the time of PASH syndrome diagnosis. Detailed demographic, clinical, and treatment information for each patient is provided in Table 1.

**Table 1:** Demographic, Clinical, and Treatment Information

Patient Information						Disease Information						Treatment Information		Relevant Laboratory Results (Before Treatment)											
Case	Age at Initial OSU Presentation	Gender	Ethnicity	BMI	Co-Morbidities	HS Locations	PG Locations	Year of Initial OSU Presentation	Year of Diagnosis (HS, PG, PASH)	Acne History (Current or prior)	PARACELSUS Score	Failed Treatments (Dose)	Effective Treatments (Dose)	Zinc (60–106 mcg/dL)	Vitamin A (38–98 mcg/dL)	WBC (3.99–11.19 K/uL)	Platelets (150–393 K/uL)	Hemoglobin (11.4–15.2 g/dL)	Calprotectin (< 50)	Anti-saccharomyces	Rheumatoid factor	ESR (< 20 mm/hr)	Colonoscopy Results	Serum Immnofixation	Pathology Results
1	30	F	White	32.6	Obesity	Back, hips, inguinal regions, left labia majora, mons pubis, posterior legs	R intergluteal fold	2014	2006 (HS), 2014 (PG), 2015 (PASH)	Current	13	Triamcinolone injections (10 mg/cc), Adalimumab (40 mg/0.8 ml), infliximab (870 mg in 09% NaCl, isotretinoin capsule (40 mg), oral doxycycline (unknown), vitamin infusion	Triamcinolone injections (10 mg/cc), Infliximab infusions (10 mg/kg q 4 weeks), Zinc, Vit A, Multivitamin infusion, excisions	37	8	6.6	310	11.8	N/A	N/A	< 10	7	N/A	Normal (no monoclonal protein present)	Perivascular and periadnexal dermatitis with associated pustular folliculitis and follicular rupture
2	35	M	White	32.8	Crohn’s disease complicated by anal fistula, renal disease, anemia, arthritis, depression	Bilateral facial cheeks, neck, XXXuttock, thighs, and lower legs	Chest, scalp, armpits, right abdomen ostomy site	2020	2000 (per patient, HS), 2019 (PG), 2020 (PASH)	Current	11	Oral doxycycline, oral clindamycin, oral rifampin, topical corticosteroids, triamcinolone injections, infliximab, adalimumab injections, prednisone, azathioprine (doses unknown)	Infliximab infusions (10 mg/kg q4w), dermabrasion, deroofing	62	62	12.25	532	11.2	N/A	N/A	N/A	> 130	N/A	N/A	Sinus tract, fibrosis, and chronic inflammation consistent with hidradenitis suppurativa
3	36	F	White	25.6	Recurrent sinus infections, MVP	N/A	Left posterior shoulder	2016	2012 (HS), 2016 (PG), 2017 (PASH)	Current	11	Oral doxycycline (dose unknown), topical corticosteroids (dose unknown), oral ciprofloxacin (500 mg daily), surgical drainage	Adalimumab injections (40 mg/0.8 ml), cyclosporine (100 mg twice daily), triamcinolone injections (10 mg/cc), minocycline (100 mg), clindamycin-benzoyl peroxide gel	N/A	N/A	7.62	290	9.9	N/A	N/A	N/A	N/A	N/A	N/A	Fragments of skin with spongiosis, dermal fibrosis, and mixed inflammatory infiltrate
4	37	F	White	22.6	Crohn’s disease, fibromyalgia, MS, childhood asthma, anxiety	Labia majora, labia minora, mon pubis, perineum	Peristomal	2020	2019 (HS), 2020 (PG), 2020 (PASH)	Prior	11	Adalimumab (dose unknown), broad-spectrum antibiotics (dose unknown)	Ustekinumab (90 mg), Infliximab (7.5 mg/kg), topical clobetasol (0.05% ointment BID), prednisone taper	N/A	N/A	18.05	503	9.6	39	N/A	N/A	52	N/A	N/A	Benign squamous lined tissue with focal ulceration and underlying stromal fibrosis with patchy acute and chronic inflammation, focus suggestive of fistula tract
5	47	M	African American	23.5	Weight loss, new-onset microcytic anemia	Bilateral inguinal, pubic, scrotal, and suprapubic areas	Left neck, anterior chest	2018	2018 (HS), 2018 (PG), 2018 (PASH)	Prior	11	Prednisone taper (8-week taper starting from 60 mg daily), broad-spectrum antibiotics (unknown)	N/A	23	N/A	14.32	447	6.9	< 15.6	22.9	N/A	84	Non-bleeding internal hemorrhoids, otherwise normal	Polyclonal hypergammaproteinemia, no monoclonal protein present	Dermal edema, no neutrophilic infiltrate
6	18	F	White	24.3	MDS transformed to AML	Axillae, inguinal folds	Right inguinal fold	2016	2015 (HS), 2016 (PG), 2016 (PASH)	Current	11	N/A	Treatment of AML (allogenic stem cell transplant, chemotherapy)	61	24	1.4	24	9.5	N/A	N/A	< 13	71	N/A	Normal (No monoclonal protein present)	N/A
7	31	F	African American	38.8	RA, HTN	Axillae, mons pubis	Bilateral lower extremities	2014	2012 (HS), 2012 (PG), 2014 (PASH)	Current	14	Prednisone tapers	Adalimumab injections (40 mg/0.8 mL)	N/A	N/A	6.1	449	7.4	N/A	N/A	< 20	130	N/A	Normal (no monoclonal protein present)	N/A
8	59	M	White	29.3	Buried penis	Arms, right hand, groin folds, perineum, buttocks	Umbilicus	2020	2016 (HS), 2020 (PG), 2020 (PASH)	No known history	13	Topical antibiotics, clindamycin (doses unknown)	Adalimumab injections, prednisone, surgical reconstruction	N/A	N/A	13.03	486	9.6	15	< 20	< 10	21	N/A	N/A	Cutanaeous ulceration with polypoid granulation tissue, acute and chronic inflamation and fibrosis

MVP = mitral valve prolapse, MS = multiple sclerosis, MDS = myelodysplastic syndrome, AML = acute myeloid leukemia, RA = rheumatoid arthritis, HTN = hypertension

#### Patient 1

**Fig. 1:**
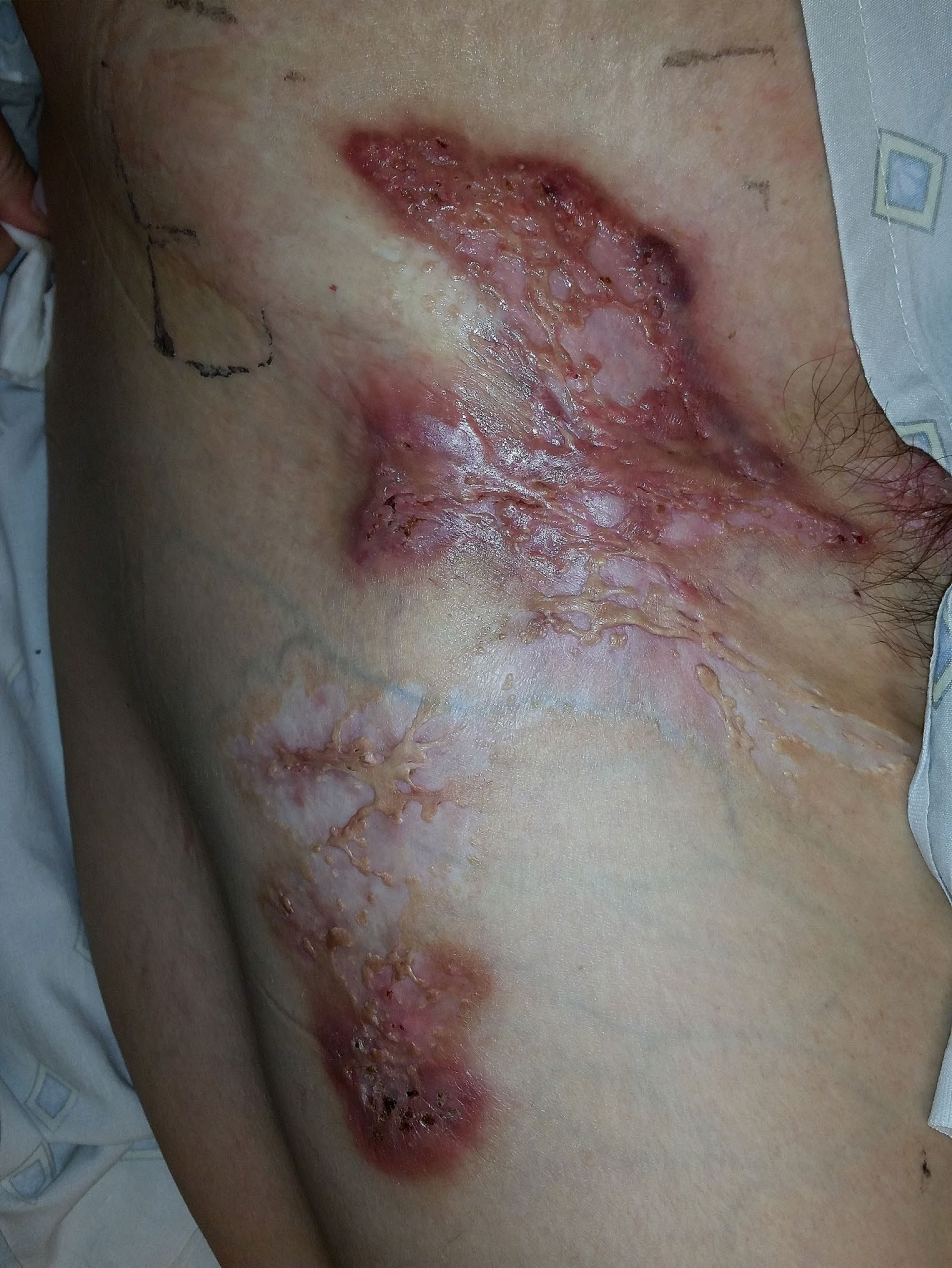
Inflammatory ulceration with extensive cribriforming and superficial tunneling in a patient with chronic severe hidradenitis suppurativa and within a region normal for hidradenitis suppurativa

A 30-year-old white female with obesity, acne, remote biliopancreatic diversion for obesity, and a 9-year history of HS presented to our clinic with a chief complaint of worsening HS on her right hip, vulva, and buttocks. Prior management for her dermatologic conditions included multiple types of oral antibiotics including doxycycline, oral isotretinoin, triamcinolone injections, and one incision & drainage for the right labia. Initial physical exam was notable for erythematous tender nodules, superficial ulcerations with cribriform appearance with overlying crust and drainage, sinus tracts, and scarring (Fig. 1). Based on the clinical appearance and history of acne, a clinical diagnosis of PASH syndrome was made. The patient was continued on intralesional triamcinolone injections and was started on high-dose, high frequency infliximab infusions in early 2016. While this led to significant improvement in her PG ulcers, her HS lesions remained persistent, and additional laboratory workup revealed a zinc and Vitamin A deficiency. Oral supplementation of these vitamins and minerals did not significantly improve her PASH syndrome; however, the recent addition of zinc and vitamin A infusions in combination with 10 mg/kg infliximab every 4 weeks infusions has improved her HS and resulted in the ability to taper her triamcinolone injections.

#### Patient 2

A 35-year-old white male with Crohn’s disease, renal disease, HS, and PG presented to our clinic with a chief complaint of persistent poorly controlled HS and PG. He had previously tried infliximab, adalimumab, prednisone, triamcinolone injections, topical corticosteroids, chlorhexidine washes, benzoyl peroxide washes, and azathioprine with limited improvement. Initial physical exam was notable for scarred cribriform erythematous plaques with sinus tracts on the bilateral facial cheeks, neck, buttocks, and thighs and lower legs, inflammatory subcutaneous nodules on the chest, surgical scars on the scalp and armpits, and an ostomy on the right abdomen. His PASH syndrome was ultimately effectively treated with combination of high-dose, high-frequency infliximab and repeated surgical debridement and dermabrasion for his residual scarring [9]. He remained on infliximab during his procedures and did not suffer from pathergy.

#### Patient 3

A 36-year-old white female with cystic acne and recurrent sinus infections presented to our clinic for a second opinion of lesions on her back that had been diagnosed as HS and PG at outside hospitals. Her dermatologic conditions had previously been managed with doxycycline, topical corticosteroids, ciprofloxacin, and incision and drainage with minimal improvement. Initial exam was notable for cystic acne on the head and face and two ulcers on the left posterior shoulder. A clinical diagnosis of PASH syndrome was made and the patient was started on adalimumab and cyclosporine. Over the past seven years, her disease improved significantly with adalimumab, with flares occurring primarily when the patient was unable to obtain adalimumab.

#### Patient 4

A 37-year-old white female with a history of Crohn’s disease treated with colectomy with ileostomy and proctectomy, perianal and vulvar HS, and peristomal PG was admitted for purulent drainage of fistulas and increasing pain and erythema. The dermatology team was consulted for management of her skin lesions. Initial physical exam was notable for swelling erythema, scarring of labia majora and minora, mons pubis, and perineum with draining nodules and ulcers; peristomal erythema and several ulcers around the right ileostomy site, several with purulent drainage and violaceous, undermined borders. Given her active HS and PG, a diagnosis of PASH syndrome was made and patient was recommended to restart infliximab, which she had previously tried and stopped due to social barriers to regular care. Due to travel and adherence concerns with infliximab, a questionable history of multiple sclerosis, and severe cutaneous disease, the patient was instead started on ustekinumab and has been tolerating injections every 8 weeks with improvement of her condition.

#### Patient 5

A 47-year-old African-American male with a history of recent weight loss and microcytic anemia was admitted for a large open neck wound and multiple abscesses complicated by sepsis. The dermatology team was consulted for widespread skin necrosis and wounds. Per the patient, the neck wound started as a small pimple nine months ago and had grown into an abscess. Initial physical exam was notable for indurated erythematous and hyperpigmented papulonodules, sinus tracts with drainage, and sclerotic nodules on the occiput, neck, suprapubic area, and inguinal folds, as well as multiple well-demarcated undermined ulcers with clean bases extending up the left chest wall. His skin biopsy was significant only for dermal edema, with neutrophilic infiltrate characteristic of PG absent; however, the diagnosis of PASH was clinically made by the patient’s symptoms. He was managed with chlorhexidine gluconate solution wash and clindamycin wash to his HS lesions, clobetasol ointment for his PG lesions, a prednisone taper, and calcium and vitamin D supplementation, with biologics in reserve if needed. The patient was readmitted three months later for worsening wounds and sepsis. A GI workup at the time was inconsistent with inflammatory bowel disease. An extensive malignancy workup including a bone marrow biopsy and lymph node biopsy was subsequently performed and unremarkable. The patient was lost to follow up after his second hospital admission. He died of unknown causes.

#### Patient 6

An 18-year-old white female with facial acne resistant to antibiotics and isotretinoin, and HS presenting with two months of headache, dyspnea with exertion, prolonged heavy menstrual cycles, and spontaneous leg bruising was admitted to the inpatient service for workup of pancytopenia and febrile illness. The dermatology team was consulted for her skin lesions, with initial physical exam demonstrating acneiform eruption of the face, acneiform HS in axillary folds, and violaceous ulcer of right inguinal fold consistent with PG. A diagnosis of PASH syndrome was made. Treatment involved silver dressings, twice daily clobetasol to the PG ulceration and daily clindamycin applied to the face. Later inpatient workup was significant for myelodysplastic syndrome, which transformed to acute myelogenous leukemia (AML) on repeat bone marrow biopsy. A comorbid hematologic malignancy is atypical for currently reported PASH syndrome. The patient underwent induction chemotherapy and allogenic stem cell transplant and continued recommended dermatologic treatment with added metformin and intralesional triamcinolone to HS nodules for better disease control, with other modalities like oral antibiotics, adalimumab, and dapsone contraindicated due to *Clostrioidies difficile* infection, AML, and anemia, respectively. Although dermatologic treatment stabilized the patient’s PASH syndrome, full improvement with wound healing and inflammation occurred with standard AML treatment of induction cytarabine and daunorubicin. Unfortunately, the patient later passed away due to complications of AML.

#### Patient 7

A 31-year-old African American female with PG in the right lower extremity that had since healed, HS with prior unclear surgical intervention, rheumatoid arthritis (RA), and hypertension presented as a hospital dermatology consult for a “flare” of PG and RA. She noted acutely worsening leg pain, foul odor, and itch for 2 weeks, and described a history of intermittent flares of PG requiring hospitalization. She was previously treated with prednisone tapers and doxycycline. Initial physical exam revealed bilateral lower extremity edema, multiple draining LLE ulcerations, RLE pustules, and bilateral axillae with numerous draining nodules and sinus tracts with discrete superficial mons pubis erosions. Scarce facial acne and hyperpigmented macules on the back were noted as well as joint deformity of the left hand. White blood count was 19.1 g/dl and significant infectious burden was suspected. Her clinical diagnosis was consistent with PASH or PAPASH syndrome, and piperacillin/tazobactam and vancomycin, methotrexate, chlorhexidine gluconate solution wash to the bilateral axilla, leg wound care with ¼ strength Dakins solution and xeroform, and adequate pain control was recommended. Anemia and acute kidney injury later developed, and methotrexate was discontinued. Six months later in the outpatient setting, the patient had received 2 doses of infliximab which controlled her pain and reduced PG severity, but she had infusion reactions, and HS worsened with fluctuant nodulocystic lesions. Weekly adalimumab 40 mg was initiated which led to further improvement of HS and PG.

#### Patient 8

**Fig. 2:**
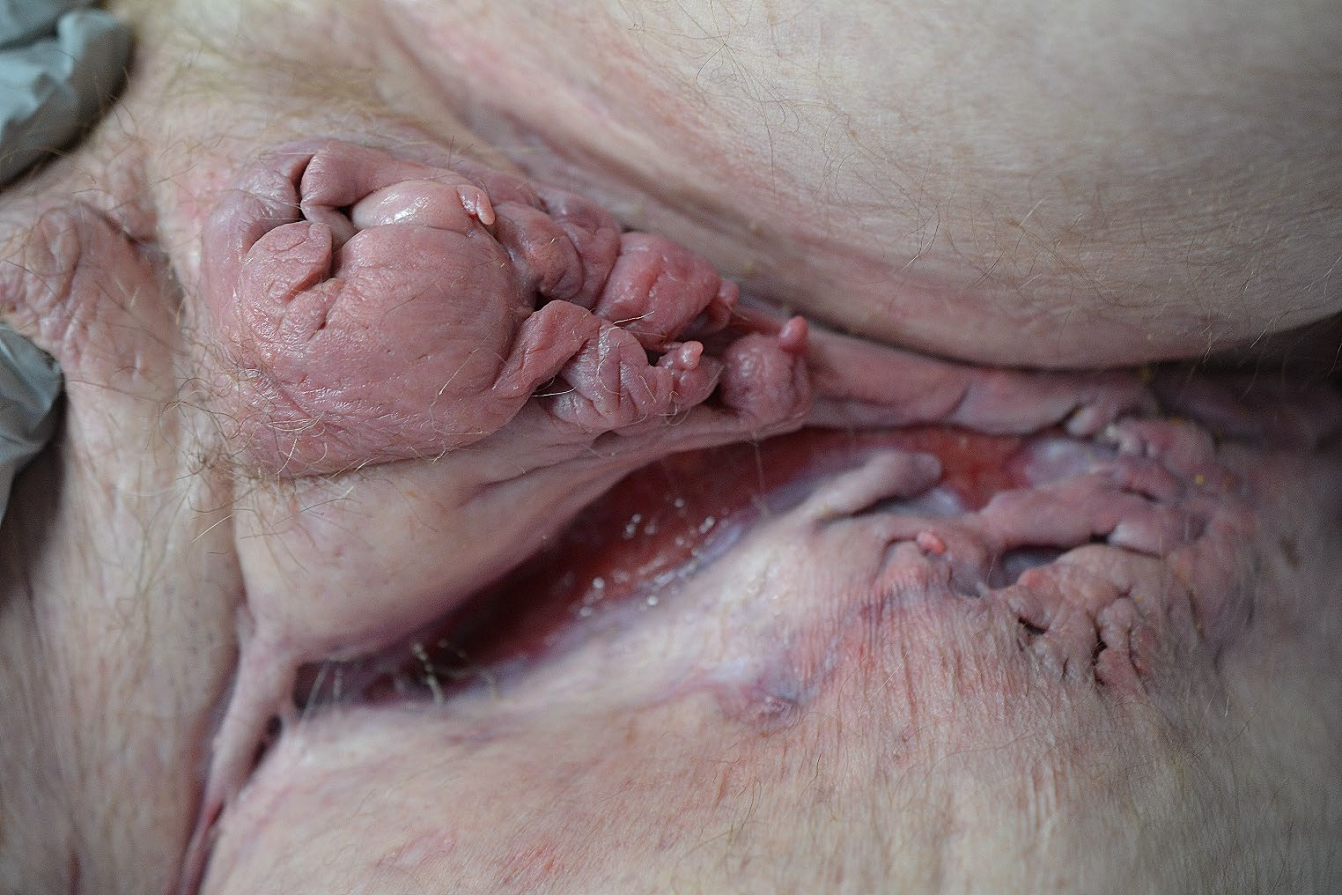
Inflammatory ulceration in a typical location for hidradenitis suppurativa with deep undermined edges and cribriform scarring, with extensive tunneling of hidradenitis suppurativa just inferior. Note that the scrotum has been obliterated by the disease

This is a 59-year-old white male patient with buried penis and previously diagnosed HS initially seen in the outpatient setting for ongoing HS. He had been managed with topical antibiotics and oral clindamycin with little success and noted a recent 30 pound weight loss. Physical exam revealed multiple sinus tracts on the bilateral arms and right hand, cribriform scarring resulting in disfiguring ulcers of the perineal anatomy, significant swelling of the inguinal region and deep tracking ulceration of the umbilicus, and erythematous papules and plaques with silver scale on the right elbow (Fig. 2). Prednisone taper and work-up for concurrent Crohn’s disease with a plan for CT abdomen and Crohn’s disease workup was initiated, but he was admitted to the hospital 10 days later for hyperglycemia and hypokalemia believed to be secondary to prednisone use. The consult dermatology team diagnosed mild HS with concurrent severe PG and recommended collaborative treatment plan with urology, plastic surgery, and colorectal surgery. After steroid initiation, he noted some lesion improvement but significant side effects. The dermatology team recommended colonoscopy to discern the underlying cause of PG, along with wound cultures and proper wound care. Prednisone dose was decreased, and at a two-month outpatient follow-up, a significant increase in PG secretions and pain was noted, although inguinal fold cribriform ulcers and purple nodule above umbilicus appeared to be healing. Adalimumab was initiated for better disease control. At later visits, the patient noted some improvement with a healthier wound bed in the scrotal skin, complete healing of the left inguinal fold, and significant re-epithelialization and webbing. The patient was lowered to 5 mg daily prednisone dosing with continued adalimumab, and the patient was lost to follow-up after surgical reconstruction for his buried penis.

## Discussion

Our study demonstrates that many different therapeutic approaches are used to manage PASH syndrome. A variety of medical treatments were tried, including oral corticosteroids, intralesional corticosteroids, oral antibiotics, and biologics. Two patients also proceeded with surgical management of their HS lesions. While treatments had varying efficacy, six out of eight patients demonstrated successful disease management defined as partial or complete closure of the initial wounds with biologics such as adalimumab, infliximab, and/or ustekinumab. Both patients with comorbid Crohn’s disease experienced significant improvement with biologics, consistent with prior studies suggesting a common pathergy between inflammatory bowel disease, PG, and HS [5, 6]. Medical management should be considered in all PASH syndrome patients without contraindications. However, some patients may experience adherence difficulties with biologics, as the frequency of infusion could pose a challenge for patients who may have professional or transportation constraints, resulting in biologic treatment failure for three patients prior to presentation at our institution. Careful counseling on the risks and benefits of initiating biologics as well as ensuring adequate social support to optimize adherence is required prior to treatment initiation.

In addition to medical treatment, vitamin supplementation can be considered in patients who are deficient. In our study, three of our eight patients were found to be deficient in either zinc, vitamin A, or both. Of these, one patient experienced disease improvement with vitamin infusions, likely related to intestinal malabsorption. Our findings are consistent with previous literature linking vitamin deficiency to HS and zinc and vitamin supplementation to disease improvement in HS [10, 11]. Lab monitoring of zinc and vitamin levels should be considered in patients with PASH syndrome and comorbid gastrointestinal conditions, such as Crohn’s disease or prior gastrointestinal surgeries.

The surgical management of debridement of facial HS in patient 2 has been described in the literature [7]. HS alone can be treated with surgical intervention in severe cases; however, concurrent PG often represents a significant risk for worsening pathergy [12]. Notably, this case series suggests careful debridement may be used to alleviate significant HS burden in these patients provided that the patient is undergoing concurrent immunosuppression for inflammation control. Optimization of medical management should be prioritized before proceeding to procedural approaches, but these techniques can be considered on a case-by-case basis for patients with refractory or severe disease.

A notable theme among these cases is the lack of significant success in dermatologic treatment until the underlying cause of PG can be uncovered and managed. Crohn’s disease, rheumatoid arthritis, and AML were drivers of PG in cases, although no cause was discovered in several patients. Tailoring patient care towards an underlying condition (i.e. systemic biologics for Crohn’s disease and chemotherapy for AML), if present, is required to effectively treat PASH syndrome.

Although active acne lesions were exhibited by some patients in this case series, the focus of diagnosis and management among these PASH patients was consistently their PG and HS. The presence of acne is required for clinical diagnosis of PASH but even in cases of severe cystic acne, dermatologic treatment centered on alleviation of PG and HS symptoms. Significant inflammatory and nodulocystic acne is known to contribute to scarring, but HS and PG are often more debilitating cutaneous diseases that require acute management by a dermatologist. Five of our eight patients had an official diagnosis of acne around the time of their PASH syndrome diagnosis, two endorsed a history of acne but had no current flares, and one patient denied any acne history. More research studying the significance of acne in diagnosis and management of PASH syndrome should be undertaken.

Based on our patient results, most of our patients presented with a combination of HS and PG as the most active skin disease at the time of PASH syndrome diagnosis. For disease improvement, most patients required a combination of therapeutics with anti-inflammatory and immunosuppressive properties. As a therapeutic ladder, adalimumab, a FDA-approved therapy for HS, was successfully in a minority of patients and a reasonable first-line option. However, when this fails or for more severe patients, high-dose, high-frequency infliximab was required to achieve disease control, often in combination with localized and adjunctive treatments such as intralesional corticosteroids, systemic corticosteroids, and micronutrient supplementation.

This study is limited by the small sample size and inability to stratify patients into different groups to analyze therapeutic benefits and outcomes. Furthermore, our patients are predominantly Caucasian, which may not be representative of the global epidemiology of PASH syndrome. Additional investigation with a greater number of patients is required to further characterize clinical characteristics and develop therapeutic guidelines for disease management. Finally, none of the patients in this case series were referred for genetic testing and counseling. As PG, acne, and HS can be seen in other autoinflammatory conditions, alternative diagnoses cannot be definitively excluded without more precise and validated case definitions [13].

## Conclusion

This case series describes the largest number of PASH patients in the literature to date and provides useful information regarding effective and ineffective treatments for this condition as well as clinical associations. Future studies should seek to evaluate a larger number of patients across multiple institutions to better control for confounding, determine significant associations in this patient population, and develop management guidelines for this condition.

## Data Availability

No datasets were generated or analysed during the current study.
